# Etched 3D-Printed Polycaprolactone Constructs Functionalized with Reduced Graphene Oxide for Enhanced Attachment of Dental Pulp-Derived Stem Cells

**DOI:** 10.3390/pharmaceutics13122146

**Published:** 2021-12-13

**Authors:** Austin J. Bow, Thomas J. Masi, Madhu S. Dhar

**Affiliations:** 1 Department of Large Animal Clinical Sciences, College of Veterinary Medicine, University of Tennessee, Knoxville, TN 37931, USA; mdhar@utk.edu; 2 School of Medicine, University of Tennessee Graduate, Knoxville, TN 37920, USA; tmasi@utk.edu

**Keywords:** tissue engineering, biomaterial, polycaprolactone, reduced graphene oxide, functionalized, dental pulp, stem cells, multi-potent, 3D printing, scaffold

## Abstract

A core challenge in the field of tissue engineering is the ability to establish pipeline workflows for the design and characterization of scaffold technologies with clinically translatable attributes. The parallel development of biomaterials and stem cell populations represents a self-sufficient and streamlined approach for establishing such a pipeline. In the current study, rat dental pulp stem cell (rDPSC) populations were established to assess functionalized polycaprolactone (PCL) constructs. Initial optimization and characterization of rDPSC extraction and culture conditions confirmed that cell populations were readily expandable and demonstrated surface markers associated with multi-potency. Subset populations were transduced to express DsRed fluorescent protein as a mechanism of tracking both cells and cell-derived extracellular matrix content on complex scaffold architecture. Thermoplastic constructs included reduced graphene oxide (rGO) as an additive to promote cellular attachment and were further modified by surface etching a weak acetic acid solution to roughen surface topographical features, which was observed to dramatically improve cell surface coverage in vitro. Based on these data, the modified rGO-functionalized PCL constructs represent a versatile platform for bone tissue engineering, capable of being applied as a standalone matrix or in conjunction with bio-active payloads such as DPSCs or other bio-inks.

## 1. Introduction

Biomaterials stand as a cornerstone of tissue engineering and represent a key means for providing structural architecture in volumetric tissue injuries. Given that native tissue varies in both mechanical and cellular properties based on location and function within the body, it is important for biomaterials to resemble or compliment these characteristics to facilitate a conducive environment for repair [1]. In developing such a platform, it is advantageous to utilize material components that have previously demonstrated biocompatibility and readily allow for cellular attachment. Polycaprolactone (PCL) is a commonly utilized thermoplastic in tissue engineering, with applications across multiple tissues, and is approved by the FDA as a biodegradable polymeric material for implantation [2,3,4,5,6]. A multitude of publications have described the application of PCL-based constructs in both in vitro and in vivo settings, commonly utilized in tissue engineering strategies geared toward bone repair. Particularly in pure PCL structures generated through heat extrusion, smooth surface topography can result in reduced capacity for cellular adhesion observed during the printing stage [7,8,9]. To circumvent this, solvents can be incorporated during the printing process to produce constructs with increased surface roughness [10]. As one of the common solvents for PCL, acetic acid has been implemented in a number of studies that use either extrusion printing or electrospinning methods for depositing the polymer onto a substrate [11]. However, solvents appear to most often be applied to form pre-printing solutions, which can complicate printing parameters, such as requiring freezing temperatures for a printing surface, and increase safety hazards to printer operators due to fume production [12,13]. As an alternative to this process, we have opted to implement a post-printing weak acetic acid washing step, allowing for surface etching to enhance cellular attachment without interrupting the streamlined printing parameters for low molecular weight PCL. Despite stability as a structural support in non-weightbearing conditions, PCL has been observed to be a relatively inert biomaterial and may require more bio-active additives to facilitate effective injury repair [14]. Furthermore, the thermoplastic exhibits an extended degradation period, ranging from 1–2 years, and can therefore present a barrier for tissue ingrowth in some cases [15,16]. To address this, the application of lower molecular weight PCL plastics and the addition of additives, such as chitosan, nano-hydroxyapatite, and graphene, have been investigated [17,18,19,20].

Graphenic materials represent a promising and versatile additive for the field of tissue engineering, with a wide array of forms and chemical modifications. Most prolific in recent biomaterials designs are the forms graphene oxide (GO) and reduced graphene oxide (rGO), both of which offer potential as delivery vehicles for biological therapeutics or as bio-active surface coatings [21]. Of these, rGO, which results from the reduction of GO by either chemical or microwave methods, has been demonstrated to be both biocompatible and to present osteo-inductive activity in vitro [22,23]. This phenomenon was particularly evident when implementing rGO, in which ascorbic acid was utilized as the reducing agent, with rGO/PCL meshes stimulating calcium deposition and increases in osteogenic gene expression [24]. For these reasons, rGO was blended with PCL using dimethyl sulfoxide (DMSO) to enhance the basal thermoplastic constructs.

Incorporation of hydrogels within rigid thermoplastic scaffolds offers a means for generating constructs that are both mechanically stable and boast bio-active features, either due to the hydrogel itself or encapsulated elements. Hydrogels represent a broad category of hydratable materials that are capable of setting, in either a permanent or reversible reaction, to a solid or semi-solid form [25,26]. Pluronic F-127 (PF127), Poloxamer 407, is a hydratable tri-block copolymer that forms a thermosensitive aqueous solution with reversible gelling characteristics. Commonly utilized at solution concentrations of 20–40% *w*/*v*, PF127 exhibits liquid characteristics at low temperature, >10 °C, and increases in viscosity as temperatures approach room temperature, gelling fully to form stable structural patterns [27]. Due to its unique gelling characteristics, PF127 has observed applications in 3D printing as a sacrificial ink and as a mechanism for encapsulating therapeutic elements [10,28]. However, studies to examine PF127 gels alone as a potential delivery vehicle for cell-based therapeutics revealed that encapsulation of cells within gel bodies experienced cytotoxic effects due to restrictive nutrient transfer [29]. Therefore, our interest in PF127 focused on its application as a coating to enhance cellular attachment to PCL architecture or to serve as a carrier for cell-derived products as compared with live cell populations.

A critical obstacle in the development of novel biomaterials is demonstrating how the material interacts with its surrounding environment following implantation in the complex and dynamic system that is the body. Though limited in direct translation, in vitro culture can lend valuable insight into the basic interactivity of a construct with cells. Moreover, the parallel development a stem cell line alongside the fabrication process for a biomaterial offers the potential to streamline the in vitro assessment process, as this provides a ready source of characterized primary cells for application at various stages of the design process for a material. For this reason, we explored methods for extracting, expanding, and characterizing populations of multi-potent cells. These cells could then be employed either as a trackable biological additive for in vitro assessment of biomaterials with complex architecture or as a bio-additive for modified thermoplastic scaffolds designed for bone tissue engineering. The combinatorial use of bio-additives, such as cells or matrix proteins derived from cells, and biomaterials represents a core focus for the field of regenerative medicine. As such, the side-by-side development of these primary stem cell populations and biomaterial skeletal construct designs, which can be loaded with a biological payload, presents a versatile platform for establishing novel therapeutic systems that are readily scalable.

The regenerative potential of stem-cell-based therapies has long been realized and presented an ideal bio-active candidate for use in conjunction with thermoplastic skeletal constructs. The use of stem cells derived from adult tissue has garnered increased interest over alternative sources; however, reprogramed cell lines such as induced pluripotent stem cells (iPSCs) face substantial challenges relating to oncogenesis in vivo [30,31,32]. Though isolation of primary mesenchymal stem cell (MSC) cultures from both bone marrow and adipose tissue has been well documented, these MSCs are limited in their innate potential to undergo maturation outside of mesodermal lineages, thereby restricting their versatility as a bio-active regenerative agent [33,34]. As such, dental pulp tissue represents a potentially superior source for the development of multi-potent cultures since these cells are of neural crest origin and theoretically capable of a more robust array of differentiation lineages, including odontogenic, angiogenic, and neurogenic potential [35,36,37,38]. For this reason, dental pulp stem cells (DPSCs) have become an attractive source for generating adult multi-potent cell cultures to be utilized as a bio-additive in a variety of applications. These range from traditional cell culture models, examining cytocompatibility and cellular response to materials or reagents, to more clinically translatable studies focused on developing tissue engineering strategies, with DPSCs as a standalone therapeutic or to be used in conjunction with a biomaterial [39].

As with other tissues, the literature describing extraction, isolation, and expansion protocols for developing DPSCs cultures can vary greatly, with the most common source tissue either being human or rat-origin. Human-derived dental pulp, while offering a promising allographic bio-active therapeutic, can present challenges relating to immunogenicity [40,41]. Furthermore, there may be regulatory thresholds for acquiring and culturing human cells that may be prohibitive for extensive biobanking of these populations. Conversely, rat-derived dental pulp offers a rich multi-potent cell source without some of these restrictive features, though the resulting xenogeneic product may not be as potent as a human-based counterpart. Particularly for research applications such as biomaterial design, the ability to rapidly develop cultures from rat mandibular tissue offers an attractive alternative and promotes the reduction in tissue waste from animal-based studies. For this reason, rat DPSC (rDPSC) cultures were determined to be optimal for developing in parallel with thermoplastic scaffold technologies.

Apart from species selection, there are multiple methods by which multi-potent cell populations are isolated from the origin tissue. These primarily fall into enzymatic and non-enzymatic options, with collagenase I and II being the most common enzymatic mechanisms for separating cells from extracellular matrix structures. Though effective for isolating cells from bulk tissue, enzymatic methods may have detrimental effects on cellular membrane integrity depending on exposure concentration and time. To minimize the potential damage or alteration to these cells, non-enzymatic methods focus on cell out-growth from bulk tissue after mechanical dissection, frequently through tissue mincing with a sterile blade, to disperse the tissue surface area over the culture space. This approach embraces the concept of minimal manipulation and ensures that primary passage cultures maintain supportive cell sub-populations and the protein-rich extracellular matrix. After permitting initial cell out-growth, standard trypsin-based cell passaging not only allows for the redispersal of cell clusters into a more uniform surface distribution, but also releases cells growing three-dimensionally on bulk tissue, resulting in expanded collection numbers. To better examine the differences in these methods, as well as the impact of growth medium content, for generating rapid, healthy, and scalable rDPSC populations, we compared various collection and expansion protocols. These cells were then applied to fabricated constructs to assess cellular attachment and viability on complex thermoplastic skeletal architecture that could lend to the applicability of the biomaterial platform for in vivo use.

## 2. Materials and Methods

### 2.1. Establishing rDPSC Lines

Euthanized rats were obtained following the conclusion of terminal IACUC approved studies. Mandibles were excised, scraped of excess muscle, and submerged in 70% ethanol to maintain sterility during transport to culture hood conditions. In a biosafety cabinet under laminar flow, the dental pulp canal was exposed on both the buccal and lingual faces of each hemi-mandible using a scalpel. A sterile dental probe was then inserted at the exposed port on the lingual face and moved along the canal, extruding the target tissue from the port on the buccal face. Pulp tissue was collected in a 60 mm culture dish and minced prior to allocation to one of two isolation methods. For tissue out-growth isolation (TG), minced tissue was directly added to a T25 culture flask and either α-MEM or DMEM/F12 growth media supplemented with 1% penicillin/streptomycin and 20% fetal bovine serum (FBS) was added. For tissue undergoing enzymatic isolation (ENZ), minced tissue was subjected to a 390 U/mL Collagenase-I solution and placed at 37 °C for 20 min with periodic agitation. Digested tissue was centrifuged at 300× *g* for 5 min to form a cell pellet, which was then isolated, resuspended in the same growth media described above, and deposited into a T25 culture flask. Cells were cultured at 37 °C and 5% CO_2_, with media replaced every 2–3 days to ensure fresh nutrients for populations.

Populations were observed under brightfield microscopy daily to monitor proliferative expansion and passaged upon confluency. Cells collected from passaging of these primary culture T25 flasks were transferred to T175 flasks at 5 × 10^5^ cells/flask for continued expansion, with supplement FBS being reduced to 10%. This expansion was carried out until cells reached their fourth passage, at which point enzymatically collected cells were aliquoted and cryo-banked to preserve low passage characteristics. Cryo-banking was facilitated by resuspending isolated passage pellets in a freezing solution that contained 45% unsupplemented growth media, 50% FBS, and 5% DMSO. These samples were allowed to gradually freeze at −80 °C and then transferred to liquid nitrogen for long-term storage.

In preparation for cell characterization and biomaterial cytocompatibility studies, cryo-banked aliquots were rapidly thawed and added to T175 culture flasks. Upon confluency, these cell populations were collected and distributed to respective experimental set-ups. For flow cytometric assessment of surface markers relating to stemness, 1 × 106 cell aliquots were stained with antibodies listed in Table 1, in accordance with manufacturer’s instructions, and then fixed in 4% paraformaldehyde (PFA)/PBS. This stemness marker panel was then evaluated using a FACSCalibur™ (BD Biosciences, Erembodegem, Belgium) flow cytometry system set to capture 30,000 events and gaited based on an unstained control sample. Post-processing of data was performed with FlowJo (Version 10, BD Biosciences, Ashland, OR, USA, 1996) software to generate quantitative values and normalize peaks.

Trilineage assessment was performed by seeding 6-well tissue culture plates with 2 × 10^5^ cells/well. At approximately 80–85% confluency, wells designated for osteogenic, chondrogenic, and adipogenic differentiation were exposed to growth media containing lineage-specific induction additives, listed along with respective concentrations in Table 2. Each plate contained undifferentiated control wells for staining comparisons. Based on previous work and published literature, cells were fixed for staining at 21–28 days for osteogenic plates, 14–21 days for chondrogenic plates, and 10–12 days for adipogenic plates using 4% PFA/HBSS at room temperature for 10 min and stored in HBSS. Evaluative staining was carried out for trilineage samples using Alizarin Red for osteogenesis, Alcian Blue for chondrogenesis, and Oil Red O for adipogenesis. Specifically, mineral deposition was determined by exposure to 2% Alizarin Red solution for 30 min on a plate shaker, washed twice with DI water to clear excess staining solution, and then washed with PBS to develop the pH-sensitive stain. Proteoglycan distribution was assessed by rinsing wells with a 1% acetic acid solution for 5 min, followed by Alcian Blue 2.5 pH solution (American MasterTech: Cat# STABL2.5100, Lodi, CA, USA) for 15 min on a plate shaker, and lastly washed thoroughly with HBSS to remove excess dye. Lipid content was examined by rinsing with a 60% 2-propanol solution for 5 min, then incubating with an Oil Red O working solution in 2-propanol for 10 min, before rinsing with tap water to clear excess stain. For both Oil Red O and Alizarin Red stains, counterstaining with hematoxylin for 1 min was performed to highlight cell nuclei during imaging. Stained wells were observed under either brightfield, for Alizarin Red and Alcian Blue stains, or phase contrast, for Oil Red O staining, at 5×, 10×, 20×, and 40× magnifications on a DMi1 microscope (Leica, Wetzlar, Germany). High resolution images were captured with a fitted MC 170 HD camera (Leica, Wetzlar, Germany).

Proliferation studies were conducted in 12-well tissue culture plates seeded with 5 × 10^4^ cells per well. For quantitative assessment at study time points, Alamar Blue staining reagent was added at a 1:5 ratio of media volume in sample wells and incubated at 37 °C for 4 h. Data was collected at 1, 3, and 5 days of growth following initial cell seeding. Plotted data points were then assessed for linearity over the time course.

### 2.2. Transduction of rDPSCs with DsRed

To facilitate transduction of rDPSC cultures, lentivirus encoding DsRed was generated. Briefly, plasmids pRSV/Rev (Addgene #12253, Watertown, MA, USA), pMDLg/pRRE (Addgene #12251, Watertown, MA, USA), pMD2.g (Addgene #12259, Watertown, MA, USA), and pLenti CMV Neo Dest (Addgene #17392, Watertown, MA, USA) encoding a DsRed open reading frame were mixed at a 1.25:1.25:6.25:6.25 ratio along with polyethylenimine (PEI) (MW, Polysciences, Inc, Warrington, PA, USA) at a 3:1 (PEI:Plasmids) ratio in unmodified DMEM media for 20 min. Media containing the DNA/PEI complexes and supplemented with 5% FBS was then added to 2.5 × 10^7^ HEK293 T/17 cells (ATCC, Manasses, VA, USA) at 24 h post-plating. Cells were incubated with the transfection media for 4 h at 37 °C and 5% CO_2_, before being replaced with DMEM media containing 5% FBS. Forty-eight hours after transfection, lentivirus-rich media was removed and centrifuged at 600× *g* then passed through a 0.45-μm filter to clear cellular debris. To concentrate the lentivirus, the solution was added to Oak Ridge centrifuge tubes, underlaid with 20% sucrose/PBS, and spun for 4 h at 20,000× *g* [42]. The resulting viral pellet was resuspended in PBS, aliquoted, and stored at −80 °C.

To titer the lentivirus, 2 × 10^5^ HEK293 T/17 cell suspensions in DMEM media containing 5% FBS were mixed with 8 μg/mL hexadimethrine bromide (polybrene) (Sigma-Aldrich, St. Louis, MO, USA) and varying volumes of concentrated lentivirus, before plating to a 6-well tissue culture plate and incubating for 48 h. After media replacement and a further 48 h of culturing, cells were enzymatically harvested with trypsin and fixed in 4% PFA/PBS for 10 min. Transduction efficiency was then determined via flow cytometry, using the excitation/emission (556/586 nm) characteristics of DsRED. The infection titer was calculated for wells displaying 5–30% positive fluorescent population based on established formulaic methods [43].

Transduction of DMEM/F12-cultured rDPSC populations was conducted by exposing 1 × 10^6^ cells suspended in growth media containing 10% FBS and 1% penicillin/streptomycin to 8 μg/mL polybrene and DsRED lentivirus at a multiplicity of infection (MOI) of 10, based on previous efficiency data for rat mesenchymal stem cells (MSCs). Cells were then plated to a T175 culture flask and incubated for 48 h before replacing the media with fresh growth media supplemented with 20% FBS. Cell culture expansion and cryopreservation were then carried out as earlier described. Fluorescent imaging of transduced populations, hence forth designated as rDPSC-R, was performed and overlaid with phase contrast captures to verify population percentages expressing the DsRed.

### 2.3. Biomaterial Design and Fabrication

To examine the application of rDPSC populations as a bio-additive for biomaterial technologies, constructs composed of either a rigid thermoplastic or thermosensitive gel were designed and fabricated. Rigid structures were comprised of heat-extruded 14,000 M_W_ PCL or a blended mixture of PCL with 1% rGO additive, synthesized by the Hummers’ method and reduced using the microwave method (Cheap Tubes), using DMSO as a solubilizing agent at 1 mL:1 g (DMSO:PCL) and heating to 60 °C. The blended PCL-rGO mix was then added to a 3 mL syringe tube and cooled to form readily available and pre-weighted material plugs. Thermosensitive gels were generated using either 30 or 40% *w*/*v* PF127 mixtures solubilized in nanopure water or DMEM/F12-based growth media. Additionally, PF127 solutions were prepared containing protein-rich ECM content from either rDPSC or rDPSC-R sources. ECM-rich additive was isolated by subjecting culture populations to 1 mM EDTA solution overnight at 4 °C to release cells [44]. After removal the cell-laden solution, the remaining attached matrix proteins were scraped, collected, and pelleted by centrifugation at 300× *g*. The resulting product was then resuspended to add to gel solutions.

Biomaterial fabrication was carried out using a BioX6 extrusion 3D printer system (Cellink, Boston, MA, USA) fitted with a thermoplastic printhead with a 0.2 mm nozzle (PCL-based constructs) and a standard pneumatic printhead with a 0.6 mm nozzle (PF127-based matrixes), with material compositions and respective print settings for this system displayed in Table 3. PCL and rGO-PCL lattice structures were designed in Autodesk Inventor CAD software to tailor interior negative space within constructs and to add sacrificial supports. Briefly, bulk scaffold mats were designed to be 20 × 20 × 2 mm (L × W × H), with a maximum negative space of 68% and a minimal pore size of 350 μm. Lateral primary layer line supports, “support wings”, were included and secured with adhesive tape following the completion of the first print layer to prevent dislocation of scaffold bodies at later subsequent layers due to a refrigerated print surface. Upon print completion, wing supports were removed and bulk material mats sectioned to four scaffolds, which were UV cured for 2 h for sterilization. To enhance surface topography of heat extrusion-based constructs, samples were “etched” by exposure to 3% acetic acid solutions for 1 h on a plate shaker, thorough rinsing with nanopure water, and air drying prior to sterilization.

Thermoplastic constructs were examined under SEM for general characterization of surface smoothness comparing PCL and rGO-PCL scaffolds with “etched” counterparts. Furthermore, adherence characteristics were assessed by seeding of DMEM/F12 cultured rDPSCs stained with 1,1′-dioctadecyl-3,3,3′,3′-tetramethylindocarbocyanine perchlorate (DiI) cytoplasmic dye at 100,000 cells/scaffold. DiI staining was carried out prior to cell seeding at 50 μg/mL per 1 × 10^6^ cells and as previously described [45]. Imaging of DiI-labeled cells was conducted up to two weeks to compare distribution and proliferative capacity of rDPSCs on thermoplastic constructs. Additionally, Calcein-AM staining was performed as previously described to verify the viability of attached cells [46]. Briefly, 50 μg of Calcein-AM CellTrace dye (ThermoFisher, Waltham, MA, USA) were reconstituted in 10 μL of DMSO to form the concentrated stock, which was then further diluted at 2 μL:1 mL of HBSS for the final staining solution. Cells were washed once with HBSS then exposed to the staining solution for 5 min at 37 °C before imaging.

Thermosensitive PF127 gel matrixes were fabricated using a generic cuboidal shape available on the printer’s DNA Studio3 software with infill lines of the solid shape constituting the resulting matrix. Printing was conducted on a surface heated to 37 °C to ensure gelation and structure integrity. Using on-board multi-well settings, gels were printed automatically and directly into a 24-well non-tissue culture plate. Finalized prints were transferred directly to cell culture incubators set to 37 °C until ready for cell addition to prevent a collapse of the matrix due to cooling at room temperature. Matrixes containing the rDPSC-R-ECM additive were assessed under fluorescence to verify content. Gels were seeded with 50,000 cells each to examine cell viability and adherence.

Modification of both PCL and rGO-PCL scaffolds with nutrient-rich PF127-ECM-R-40 gel was performed to determine whether these bio-inks improved cell attachment and viability. Briefly, skeletal constructs were dip-coated in PF127 mixtures on ice for perfusion throughout internal surfaces, transferred to sterile 24-well non-tissue culture plates, and stored at 4 °C to allow for excess solution to gradually settle out of scaffolds. Fluorescent imaging was performed to verify rDPSC-R-ECM content. Calcein-AM staining was carried out after two weeks to observe density of metabolically active cells within biomaterials.

## 3. Results

### 3.1. Establishing Rat DPSC Lines

**Figure 1:**
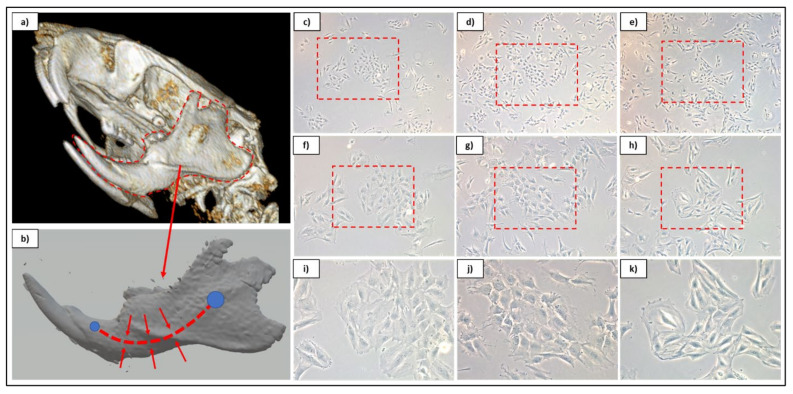
Overview graphic for dental pulp site on CT rendering (**a**) and reconstructed mandibular 3D model (**b**). Cultured cell isolates from αMEM-cultured cells from both enzymatic (**c**,**f**,**i**) and tissue outgrowth (**d**,**g**,**j**) methods and DMEM/F12-cultured cells using tissue outgrowth method (**e**,**h**,**k**). Phase contrast culture images are displayed at 5× (**c–e**), 10× (**f**–**h**), and 20× (**i**–**k**) magnifications.

**Figure 2:**
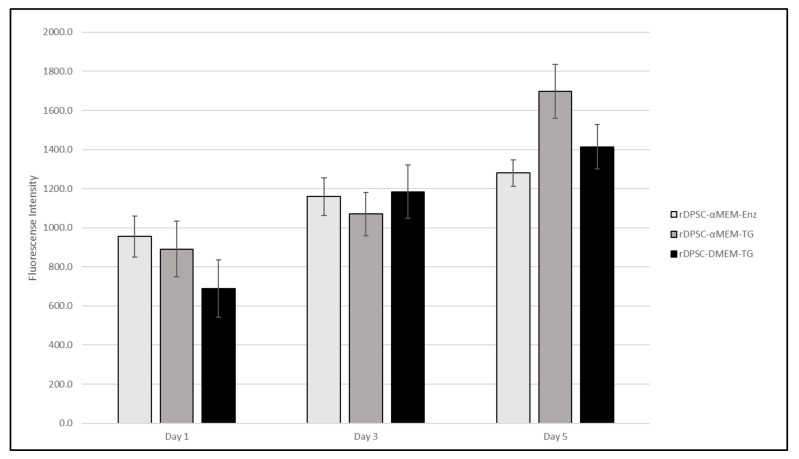
Alamar Blue proliferation data for rDPSC populations cultured in α-MEM-, both enzymatic and tissue outgrowth methods, and DMEM-based growth mediums.

Populations were observed under brightfield microscopy daily to monitor proliferative expansion and for general morphological characteristics with populations. It was noted that both of the α-MEM-cultured populations contained subset cells that demonstrated more “cobblestone” morphologies, which more resembled that of endothelial-like cells, while DMEM/F12-cultured populations maintained the spindle shape associated MSCs (Figure 1). The morphological similarities between α-MEM-cultured populations indicated that TG was a preferred methodology for collection, as this limited enzyme exposure to only during passaging. Despite this, proliferative assessment determined that all populations were capable of expansion at a similar rate, with no statistical difference observed between sample groups at time points (Figure 2). Based on these initial data, evaluation of surface markers relating to stemness was performed for both α-MEM- and DMEM/F12-cultured cells collected via the TG method, and the resulting profiles were consistent with those expected for DPSCs (Table 4).

Trilineage staining of DMEM/F12-cultured rDPSCs showed positive staining for proteoglycans and lipids for cells exposed to chondrogenic and adipogenic inductive agents, respectively (Supplementary Figures S1 and S2). Notably, calcium content was not found to increase substantially in cultures exposed to osteogenic inductive agents. Staining of α-MEM-cultured populations for comparison revealed a low level of lipid content when exposed to adipogenic media; however, cells in both chondrogenic and osteogenic media presented disrupted monolayers and unhealthy cellular morphology. Despite this, osteogenic-induced α-MEM-cultured wells did present with high levels of calcium staining, indicating cellular deposition.

### 3.2. DsRed Transduction of rDPSCs

**Figure 3:**
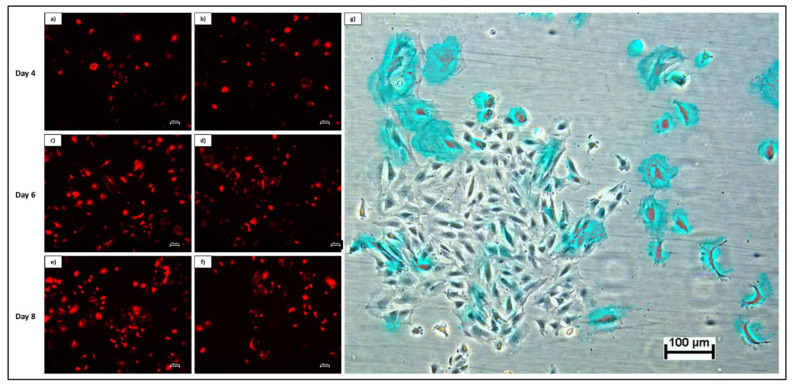
Fluorescent imaging of rDPSCs transduced with DsRed over time in the initial passage. Images at 5× magnification depict cells at day 4 (**a**,**b**), day 6 (**c**,**d**), and day 8 (**e**,**f**) of growth. Overlay of phase contrast and fluorescent imaging at day 8 show DsRed-expressing cells (shown in blue for enhanced contrast) within overall population (**g**).

Transduction of rDPSCs using DsRed-encoded lentivirus demonstrated successful integration and subsequent expression of the DsRed fluorescent protein. Merged fluorescent and phase contrast images display transduced cells within the growing population (Figure 3).

### 3.3. Biomaterial Design and Fabrication

**Figure 4:**
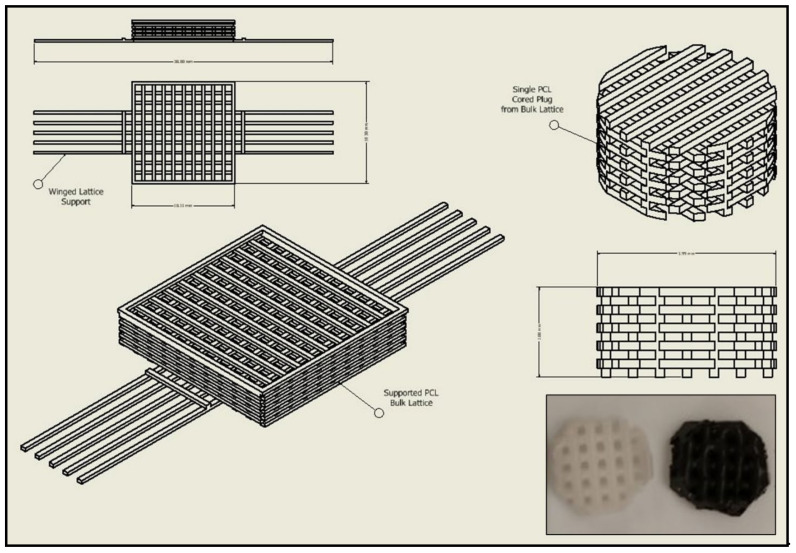
CAD schematic for print design with original structure and cored scaffold sample. Image inset depicts final printed scaffold samples of both PCL (**Left**) and rGO-PCL (**Right**).

**Figure 5:**
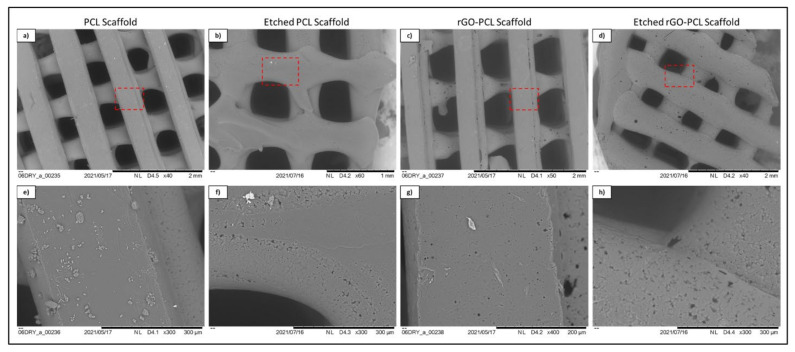
SEM imaging of etched (**b**,**d**,**f**,**h**) and unetched (**a**,**c**,**e**,**g**) PCL and rGO-PCL constructs at low (**a**–**d**) and high (**e**–**h**) magnification. Red dashed line boxes on low magnification images indicate the zone being magnified for each scaffold type.

Thermoplastic and gel-based biomaterial designs were successfully fabricated using a BioX6 (Cellink) pneumatic extrusion system (Figure 4). PCL and rGO-PCL lattice structures observed under SEM displayed highly smooth surface features and thus were exposed to 3% acetic acid solution for 1 h to allow for surface etching, which appeared as surface pitting under subsequent SEM assessment (Figure 5).

Bio-inks generated using PF127 hydrated with various aqueous solutions were conducive to automated printing in 24-well non-tissue culture plates and held form at 37 °C incubation. Upon the addition of cell solution, 30% *w*/*v* material structures appeared to rapidly lose architecture due to hydration, while 40% *w*/*v* materials maintained their composition for an extended period.

**Figure 6:**
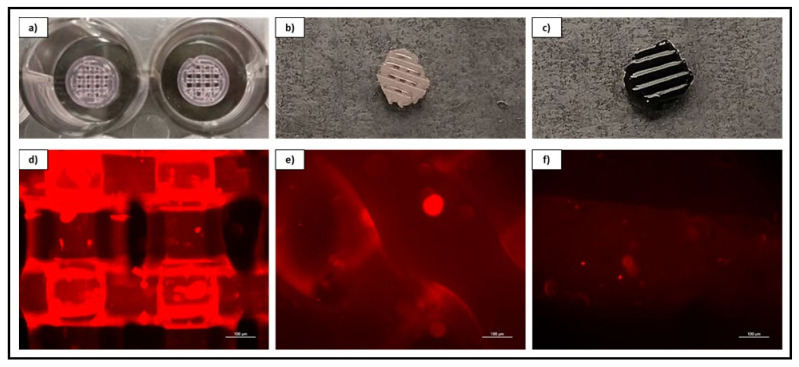
Overview and magnified (10×) fluorescent imaging of acellular 40% *w*/*v* PF-127 bio-ink containing ECM derived from rDPSC-R as a standalone printed lattice structure (**a**–**d**) and coating both a PCL (**b**–**e**) and rGO-PCL (**c**–**f**) construct.

**Figure 7:**
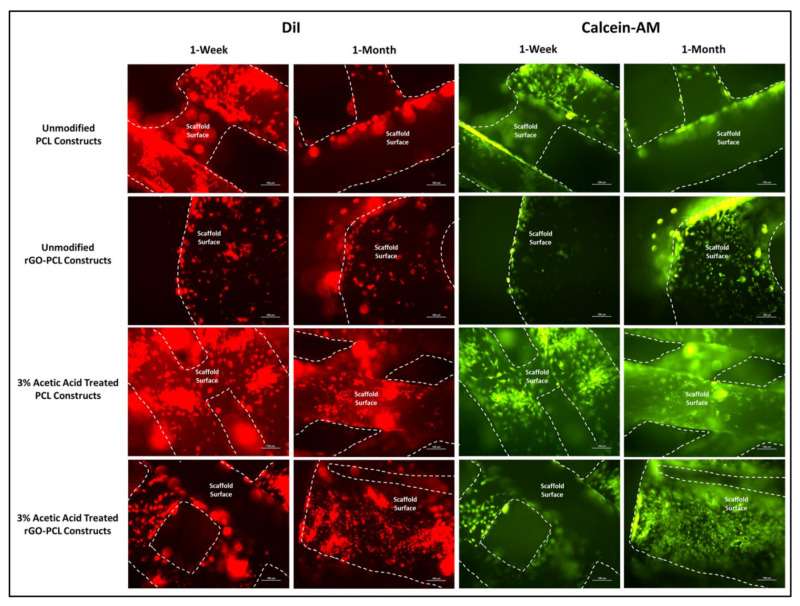
rDPSCs seeded on PCL and rGO-PCL constructs, both unmodified and etched with 3% acetic acid, under fluorescent imaging at 10× magnification. DiI-labeled and Calcein-AM-stained cells are shown with scaffold surfaces highlighted via dashed lines. Cell populations on scaffolds were assessed at both 1-week and 1-month time points.

**Figure 8:**
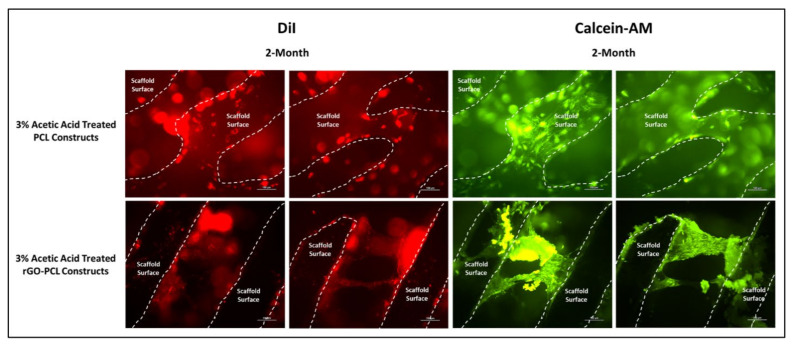
rDPSCs cultured for 2 months on PCL and rGO-PCL constructs etched with 3% acetic acid, under fluorescent imaging at 10× magnification. DiI-labeled and Calcein-AM-stained cells are shown with scaffold surfaces highlighted via dashed lines.

Fluorescent imaging of PF127-ECM-R-40 gels as standalone structures and in combination with both PCL and rGO-PCL constructs showed a clear presence of cellular protein content as displayed in red (Figure 6). This indicated that the thermoplastic basal constructs were amenable to coating with these bioactive gels. Cells seeded to PCL and rGO-PCL scaffolds were confirmed to be viable, illustrating cytocompatibility. Specifically, Calcein-AM imaging of cells on unmodified and etched constructs demonstrated that rDPSC populations were metabolically active while DiI-labeled populations gave indication of culture expansion over time (Figure 7 and Figure 8).

## 4. Discussion

rDPSC primary cell lines were developed in parallel with thermoplastic basal constructs to demonstrate a streamlined in vitro assessment process and examine their interaction of acetic-acid-etched rGO-functionalized 3D printed PCL constructs. Multiple methodologies were utilized in establishing primary cultures to elucidate a protocol that employs a minimal manipulation strategy and results in expandable populations of cells expressing characteristics associated with DPSCs. Compared with more traditional enzymatic isolation methods that utilize Collagenase-I to separate cells from bulk tissue, the use of simple tissue outgrowth from minced bulk tissue appeared to be preferrable due to both the elimination of initial enzymatic degradation and the ability for proliferating cells to expand 3-dimensionally on native structures, resulting in enhanced yields on the first passage (this was extrapolated from increased densities in TG populations 24 h following the initial passage). As determined by Alamar Blue staining, proliferative capacity of cells cultured in both mediums, including enzymatically collected populations, demonstrated similar growth profiles, indicating that all populations were amenable to expansion (Figure 2).

While both populations demonstrated surface marker data indicating stemness, there were noted morphological differences between αMEM and DMEM/F12 cultured cells, with a sub-set of cells in the αMEM populations displaying a “cobblestone” appearance that is more commonly associated with endothelial-like cells. In comparison, DMEM/F12 populations demonstrated a more uniform distribution of “spindle-shaped” cells, which are more traditionally associated with stem cells (Figure 1). Interestingly, this may indicate that the α-MEM basal media contains supplemental molecules capable of stimulating some level of differentiation within cultures, and therefore is not an ideal media for deriving multi-potent cell populations for cryo-banking, despite its use in the literature. Notably, α-MEM is utilized as the basal component in published growth mediums intended for culturing endothelial cell lines, which strengthens this observation [47,48]. This was further substantiated by trilineage differentiation attempts for α-MEM-cultured populations, in which both chondrogenic and osteogenic differentiation induction mediums resulted in monolayer disruption and unhealth cellular morphology within 7 days post-induction. It was observed that despite cell appearance, osteogenic-induced wells demonstrated substantial calcium deposition. This phenomenon may be indicative of an overstimulation of cells, since there appears to be a level of lineage induction due to growth media based on morphology [45]. Trilineage assessment of DMEM/F12-cultured populations confirmed their capacity for differentiating to chrondrocyte and adipocyte lineages, with osteogenic wells not displaying an increase in stain over control wells. Cells exposed to the osteogenic induction media maintained a healthy appearance, in contrast with α-MEM-cultured populations, and exhibited a reduction in proliferation, which would be expected for cells undergoing lineage maturation. Therefore, the lack of detected calcium deposition may be related to the concentration of inductive additives in the differentiation media. Based on these data, those rDPSC populations collected through TG methodology and expanded in DMEM/F12 were determined to be preferred for further applications as a bio-additive on PCL constructs.

To generate readily trackable cell populations, lentiviral transduction was performed on DMEM/F12-cultured rDPSC populations, resulting in fluorescent cells that could be applied to and monitored on biomaterials, confirming cell presence and density. The establishment of these fluorescent cultures further offered the potential to derive ECM protein-rich gel additives for thermoplastic skeletal constructs that were bio-active and could be observed under fluorescent microscopy, as displayed in Figure 6. Notably, cells that had been recently transduced grew substantially slower as compared with non-transduced populations at the same passage and density. This was expected to be related to viral load stress, and cells were supplemented with additional FBS to compensate [49]. Despite the increased FBS concentration, over culture time post-transduction cultures tended to see increases in the non-fluorescent cell sub-population percentage. As the cells expressing the DsRed morphologically appeared healthy, the increasing percentage of non-fluorescent cells may have simply been indicative of a more rapid proliferative capacity of cells that were not virally transduced. Alternatively, the observed subset population of low-expressing CD90 cells, indicated in Table 4, for DMEM/F12-cultured cells, may account for non-transduced cells, as CD90+ populations have been described to promote enhanced transduction potential [50,51]. Furthermore, the multiplicity of infection used for the transduction was determined based on previous transductions of rat MSCs derived from adipose and bone-marrow tissue and was anticipated to have an efficiency above 80%.

An amount of 14,000 M_W_ PCL was selected for the basal material for scaffolds due to its approval by the FDA for biomedical applications and its reduced degradation time as compared with higher molecular weight variants [52,53]. Constructs were readily fabricated via heat extrusion on a Cellink BioX6 pneumatic printer, facilitating rapid and consistent manufacture of thermoplastics lattices. As the utilized pneumatic additive manufacturing system is commercial, this may provide a more translatable set of print parameters and methodologies than observed with custom printer systems, and reduce optimization times associated with reproducing material constructs [54]. The incorporation of rGO using a simple heated blending process with DMSO removed the need for more aggressive solvents, such as commonly utilized DCM or chloroform [55,56]. This reduced cytocompatibility risks associated with residual solvents and streamlining the material synthesis protocol. Moreover, the rGO-PCL blended composite was amenable to printing under similar conditions to its PCL-only counterpart, indicating that functionalization did not dramatically alter the underlying polymeric thermal characteristics. Importantly, rGO-PCL blends were conducive to printing at structural architecture and pore size resolutions needed to facilitate ECM production and organization, with resulting dimensions similar to those in recently published literature that used alternative solvent methods [56,57,58]. SEM imaging revealed that surface topography for heat-extruded thermoplastics was highly smooth, potentially presenting a difficult attachment surface for cells. This was verified by low adherent cell numbers following seeding of unmodified scaffolds. To address this, the thermoplastic lattices were subjected to a weak acetic acid solution for one hour, which provided sufficient surface modification via etching to allow for enhanced cellular attachment and expose increased surface area of material substrates. It was noted that the use of stronger solvents, including glacial acetic acid and chloroform, resulted in substantial structural degradation and fracturing, even when applied with low volume and minimal contact time. Therefore, the application of 3% acetic acid for 1 h as post-printing modification was determined to be preferential, with Bulk ECM production by rDPSCs on these modified constructs capable of bridging larger pores by two months (Figure 8).

The inclusion of rGO as an additive in thermoplastic constructs aimed to enhance cellular attachment and health on fabricated products. Furthermore, rGO has also been previously described to promote osteogenic activity and therefore serves as an ideal component in a scaffold intended for bone tissue engineering [24]. Observation of DMEM/F12-cultured rDPSCs on thermoplastic matrices confirmed cell attachment and proliferation, with 1-week images further demonstrating increased attachment to scaffolds etched with 3% acetic acid. Interestingly, 1-month imaging revealed that both unmodified and etched rGO-PCL constructs out-performed PCL-only materials, based on greater cell numbers and surface coverage. Between rGO-PCL structures, the etched thermoplastic displayed superior cell surface coverage and health. This supported the implementation of rGO as a mechanism for improving cell-biomaterial interaction for polymeric compositions.

Thermosensitive bio-inks based on rehydrated PF127 powder demonstrated a potential to serve as a carrier for protein-rich additives that, while they may not maintain structural stability when subjected to aqueous conditions, can be coupled with a more rigid polymeric structure to enhance the intra-scaffold environment. As demonstrated in Figure 6, constructs were able to be readily coated with gel solutions, which may offer the advantage of tunable bioactive surfaces for basal constructs in future studies. Through a similar method, common tissue engineering additives, such as nano-hydroxyapatite (nHA) or tissue-specific growth factors, may be readily incorporated into a general and mechanically supportive ultra-structure, thereby allowing for a wide variety of regenerative medicine technologies to share common compositional attributes. While the lattice thermoplastic constructs assessed in this study were designed to serve as a bone tissue engineering scaffold, the skeletal architecture can be tailored based on the target tissue.

The implementation of PCL as the basal thermoplastic for structure skeletons was selected based on ease of extrusion and amenity to additives, such as rGO. Such scaffolds can be further modified by modulating the print design, in particular, alteration to the pore density and size, to develop substrates that more closely resemble the mechanical strength properties of the target tissue. Furthermore, bio-ink additives could theoretically be applied to numerous skeletal construct compositions to promote enhanced attachment or as a vehicle for a particular protein or drug compound. For example, preliminary studies were conducted in which the protein-rich additive for gels was replaced with chemically modified RNA coding for bone morphogenic protein-2 (BMP-2) with a green fluorescent protein (GFP) tag and bound to functionalized-nHA to attempt to elicit protein production (detectable via GFP synthesis). Though on-going, such substitutions demonstrate the versatility of this system to be used as an implantable construct capable of facilitating the manufacture of precise molecular compounds by either pre-loaded or endogenous cell populations.

## 5. Conclusions

Cells derived from dental pulp tissue offer a rich source for developing large scale primary cell populations to be utilized as bio-additives. Here, we describe a parallel development strategy for establishing a characterized primary cell line that can be utilized to directly evaluate an etched rGO-functionalized 3D printed PCL material at various stages of design. Comparison of extraction and culturing methods for rDPSCs indicated that cells collected through the TG method grown in DMEM/F12-based medium display preferred characteristics. We then established populations of modified rDPSCs that express DsRed, thereby allowing for a rapid and effective means of in vitro monitoring. This addresses a common complication when evaluating 3D material constructs in vitro. The fabrication of thermoplastic constructs from commercially available PCL presents a means for readily generating mechanically stable and largely inert implantable architecture that is amenable to modification with bio-active additives.

Implementation of rGO, through use of a simple heated blending with DMSO as a mild solvent, did not impact scaffold printability and was further observed to promote cell attachment and proliferation on constructs. This was further enhanced by the addition of a post-printing modification step using a 3% acetic acid to etch smooth polymeric surfaces and generate topographical sites amenable to adhesion, with superior cell surface coverage and health at one month following seeding and bulk ECM bridging of larger pores observed at two months. Our subsequent incorporation of protein-rich thermosensitive gels as a method for further functionalizing the polymeric surface demonstrated that constructs were amenable to coating with the bioactive gels. This indicated that such bio-inks may provide a versatile mechanism for fine-tuning the application of an implant for multiple tissue engineering strategies while maintaining a common ultra-structural composition. In particular, the modified rGO-PCL constructs demonstrated promise as a potential bone tissue engineering platform based on cellular attachment and expansion on substrate surfaces. For this reason, this scaffold iteration will be of pertinent interest for future in vitro experimentation to characterize gene expression profiles and metabolic activity of seeded cells to evaluate these platforms as a potential candidate for in vivo application as either a standalone scaffold or in conjunction cells or protein-rich bio-inks.

## Figures and Tables

**Table 1:** List of surface markers in characterization panel, including antibody source and respective dose per 1 × 10^6^ cells.

Surface Marker	Antibody Source	Dose (μg)
CD29	BioLegend (102219)	0.02
CD90	BioLegend (202523)	0.02
CD73	BD Pharmingen (551123)	0.05
CD106	BioLegend (200403)	1.00
CD11b/c	BioLegend (201814)	0.025
CD45	BioLegend (202211)	0.025
CD31	BD Pharmingen (555027)	0.20

**Table 2:** List of differentiation media additives including additive name and respective final concentration.

Differentiation Solution	Additive	Final Concentration
**Osteogenic**	Dexamethasone	100 nM
	Ascorbic Acid	50 μM
	β-Glycerophosphate	10 mM
**Chondrogenic**	Dexamethasone	100 nM
	Ascorbic Acid	200 μM
	TGF-β1	10 ng/mL
	Proline	40 μg/mL
	Sodium Pyruvate	1 mM
	ITS-G	1×
**Adipogenic**	Dexamethasone	1 μM
	IBMX	500 μM
	Indomethacin	200 μM
	Insulin	5 μg/mL

**Table 3:** Material identifiers, compositions, and print settings for fabricated biomaterials.

Material ID	Composition	Printer Settings			
		Printhead		Surface	
PCL	14,000 M_W_ PCL	Temperature	60 °C	*Temperature*	5 °C
		Speed	10 mm/s		
		Pressure	80 kPa		
rGO-PCL	14,000 M_W_ PCL 1% rGO DMSO	Temperature	65 °C	*Temperature*	5 °C
		Speed	10 mm/s		
		Pressure	80 kPa		
PF127-H2O-30	PF127 30% *w*/*v* Nanopure Water	Temperature	30 °C	*Temperature*	37 °C
		Speed	3 mm/s		
		Pressure	40 kPa		
PF127-Media-30	PF127 30% *w*/*v* DMEM/F12	Temperature	30 °C	*Temperature*	37 °C
		Speed	3 mm/s		
		Pressure	40 kPa		
PF127-Media-40	PF127 40% *w*/*v* DMEM/F12	Temperature	30 °C	*Temperature*	37 °C
		Speed	3 mm/s		
		Pressure	115 kPa		
PF127-ECM-40	PF127 40% *w*/*v* DMEM/F12 rDPSC-ECM	Temperature	30 °C	*Temperature*	37 °C
		Speed	3 mm/s		
		Pressure	115 kPa		
PF127-ECM-R-40	PF127 40% *w*/*v* DMEM/F12 rDPSC-R-ECM	Temperature	30 °C	*Temperature*	37 °C
		Speed	3 mm/s		
		Pressure	115 kPa		

**Table 4:** Flow cytometry data for α-MEM and DMEM/F12 cultured populations.

Surface Marker	Population Expression (%)			
	α-MEM		DMEM/F12	
CD29	94.60	+	99.50	+
CD90	97.30	+	79.70	+
CD73	77.00	+	59.40	+
CD106	4.47	-	11.00	-
CD11b/c	1.43	-	0.18	-
CD45	1.45	-	0.20	-
CD31	2.60	-	0.82	-

## Data Availability

The data presented in this study have been presented in full.

## Supplementary Materials

The following are available online at https://www.mdpi.com/article/10.3390/pharmaceutics13122146/s1, Figure S1: Brightfield imaging of Alcian Blue stained samples at 5×, 10×, 20×, and 40× magnifications, Figure S2. Phase contrast imaging of Oil Red O stained samples at 5×, 10×, 20×, and 40× magnifications.
