# Supplementary material for: Etched 3D-Printed Polycaprolactone Constructs Functionalized with Reduced Graphene Oxide for Enhanced Attachment of Dental Pulp-Derived Stem Cells
Source: Pharmaceutics. 2021 Dec 13;13(12):2146. doi: 10.3390/pharmaceutics13122146 (PMC8704510; doi:10.3390/pharmaceutics13122146)
Supplement: Supplementary file 1 [file pharmaceutics-13-02146-s001.zip › pharmaceutics-1430503-supplementary.pdf]

pharmaceutics

MDPI

Supplementary Materials: Etched 3D-Printed Polycaprolactone Constructs Functionalized with Reduced Graphene Oxide for Enhanced Attachment of Dental Pulp-derived Stem Cells

Austin J. Bow, Thomas J. Masi and Madhu S. Dhar

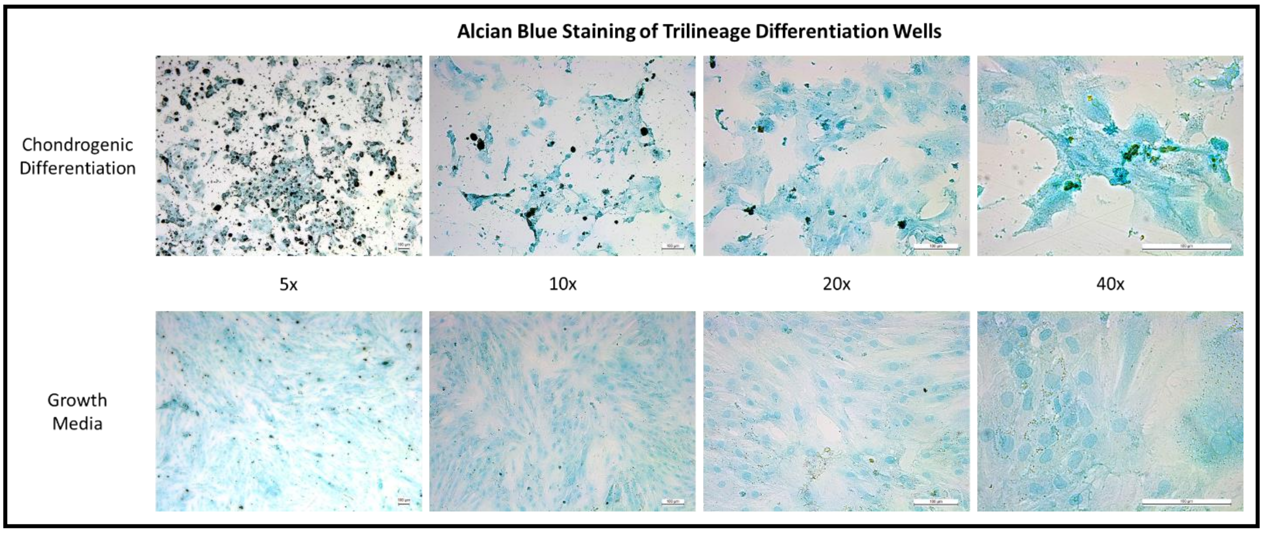

Figure S1. Brightfield imaging of Alcian Blue stained samples at 5×, 10×, 20×, and 40× magnifications.

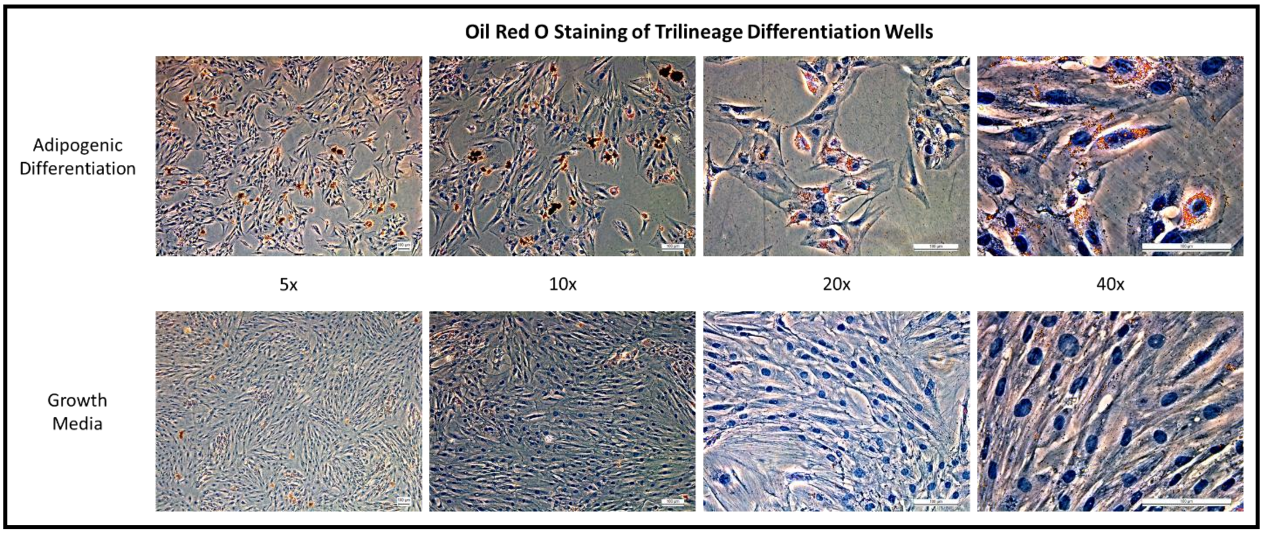

Figure S2. Phase contrast imaging of Oil Red O stained samples at 5×, 10×, 20×, and 40× magnifications.
